# Intracardiac Fibrinolysis and Endothelium Activation Related to Atrial Fibrillation Ablation with Different Techniques

**DOI:** 10.1155/2020/1570483

**Published:** 2020-02-12

**Authors:** Orsolya Hajas, Zsuzsa Bagoly, Noémi K Tóth, Réka Urbancsek, Alexandra Kiss, Kitti B Kovács, Ferenc Sarkady, Attila Nagy, Anna V. Oláh, László Nagy, Marcell Clemens, László Csiba, Zoltán Csanádi

**Affiliations:** ^1^Institute of Cardiology, Faculty of Medicine, University of Debrecen, Debrecen, Hungary; ^2^Division of Clinical Laboratory Sciences, Faculty of Medicine, University of Debrecen, Debrecen, Hungary; ^3^MTA-DE Cerebrovascular and Neurodegenerative Research Group, University of Debrecen, Debrecen, Hungary; ^4^Department of Neurology, Faculty of Medicine, University of Debrecen, Debrecen, Hungary; ^5^Department of Preventive Medicine, Faculty of Medicine, University of Debrecen, Debrecen, Hungary

## Abstract

**Objective:**

The effect of pulmonary vein isolation (PVI) on fibrinolytic and endothelial activation with currently applied periprocedural anticoagulation has not been explored. We measured markers of fibrinolysis and endothelium activation before and after PVI with the second-generation cryoballoon (Cryo), pulmonary vein ablation catheter (PVAC-Gold), and irrigated radiofrequency (IRF).

**Methods:**

Markers of fibrinolysis and endothelium activation in left atrial (LA) blood samples were measured in 31 patients before and after PVI (Cryo:10, PVAC-Gold: 7, IRF: 14). Periprocedural anticoagulation included uninterrupted vitamin K antagonist and iv heparin (ACT≥300 sec) during LA dwelling.

**Results:**

Levels of *D-dimer* (median; interquartile range, mgFEU/L) increased with all techniques (PVAC: 0.34; 0.24–0.50 versus 0.70; 0.61–1.31; *p*=0.0313, Cryo: 0.33; 0.28–0.49 versus 0.79; 0.65–0.93; *p*=0.0313, Cryo: 0.33; 0.28–0.49 versus 0.79; 0.65–0.93; *p*=0.0313, Cryo: 0.33; 0.28–0.49 versus 0.79; 0.65–0.93; *PAP complex level* (ng/ml) increased after Cryo (247.3, 199.9–331.6 versus 270.9, 227.9–346.7; *p*=0.0313, Cryo: 0.33; 0.28–0.49 versus 0.79; 0.65–0.93; *p*=0.0313, Cryo: 0.33; 0.28–0.49 versus 0.79; 0.65–0.93; *p*=0.0313, Cryo: 0.33; 0.28–0.49 versus 0.79; 0.65–0.93; *PAI-1 activity* (%) decreased with the PVAC (1.931; 0.508–3.859 versus 0.735, 0.240–2.707; *p*=0.0313, Cryo: 0.33; 0.28–0.49 versus 0.79; 0.65–0.93; *p*=0.0313, Cryo: 0.33; 0.28–0.49 versus 0.79; 0.65–0.93; *p*=0.0313, Cryo: 0.33; 0.28–0.49 versus 0.79; 0.65–0.93; *VWF antigen levels* and *FVIII activity* increased after PVI with all the 3 techniques. The levels of *soluble VCAM-1* (ng/ml) did not change after PVAC procedures, but increased after Cryo (542, 6; 428.5–753.1 versus 619.2; 499.8–799.0; *p*=0.0313, Cryo: 0.33; 0.28–0.49 versus 0.79; 0.65–0.93; *p*=0.0313, Cryo: 0.33; 0.28–0.49 versus 0.79; 0.65–0.93;

**Conclusion:**

PVI with contemporary ablation techniques and periprocedural antithrombotic treatment induces coagulation and endothelium activation of similar magnitude with different ablation methods.

## 1. Introduction

Pulmonary vein isolation (PVI) is an established therapy for the management of atrial fibrillation (AF) with a low risk of a major complication, including thromboembolism [1–3]. Available data indicate that the risk of a clinical or a silent thromboembolic event is related to the periprocedural anticoagulation routine and the ablation technology used [4–7]. The interaction between coagulation activation and the different maneuvers and techniques used during an AF ablation procedure is not fully understood. Earlier studies mostly on patients undergoing radiofrequency (RF) ablation for paroxysmal supraventricular tachycardia suggested that the coagulation cascade is activated by the introduction of any instrumentation (sheath, catheter) into the bloodstream, and conflicting results were published on the role of RF applications per se [8–11]. It has also been proposed that thrombogenesis related to catheter ablation is a 2-phase response: the acute phase during the procedure is mostly related to catheter insertion in the bloodstream and the 2nd phase is due to the tissue damage created by the ablation itself [12]. These earlier studies involved a relatively low number of RF deliveries confined to a physically limited region in the heart. On the contrary, a large area is covered within the left atrium with circumferential lesions placed around all pulmonary veins (PVs) during AF ablation, which poses a potentially more significant effect on coagulation and endothelial activation. Limited data are available on intracardiac hemostasis activation in response to the endothelial damage with contemporary AF ablation technologies and periprocedural anticoagulation routine.

Previously, our group compared hemostasis markers in blood samples obtained from the femoral vein, from the left atrium, and from the left atrial appendage in patients with AF versus in those undergoing left atrial catheterization for another substrate. The aim of this earlier work was to explore potential site-specific differences in coagulation activation as well as the differences in patients with versus without AF [13]. Herein, we measured the levels of different fibrinolytic and endothelial activation markers in left atrial blood samples before and after PVI to assess to effect of the ablation per se. Further, we compared these changes obtained with 3 different ablation technologies. These included electroanatomically guided point-by-point focal irrigated RF ablation with contact force monitoring considered by many as the “gold standard” of AF ablation, and 2 of the “single-shot” technologies: cryoablation with the 2^nd^-generation cryoballoon catheter (Arctic Front Advance) and phased RF ablation with the modified circular pulmonary vein ablation catheter (PVAC-Gold).

## 2. Methods

### 2.1. Study Population

Consecutive patients undergoing radiofrequency ablation for symptomatic paroxysmal or persistent AF were enrolled in the study based on the following: age 18–75 years, documented, symptomatic paroxysmal or persistent AF, failure of at least one antiarrhythmic drug, and willingness to sign a written informed consent. Exclusion criteria included long-standing persistent AF, reversible cause of AF (e.g. hyperthyroidism), presence of a left atrial thrombus, previous heart surgery, valvular heart disease, left ventricular ejection fraction (LVEF) ≤30%, heart failure of New York Heart Association functional classification (NYHA) class III or IV, documented carotid stenosis, history of ischemic stroke or TIA, prior cardiac surgery, unstable angina or myocardial infarction within the last 3 months, severe chronic obstructive pulmonary disease, known bleeding or thrombotic disorders, acute inflammation, contraindication to oral anticoagulation, and pregnancy.

The study design was in accordance with the guiding principles of the Declaration of Helsinki and was approved by the Institutional Ethics Committee of the University of Debrecen and the Ethics Committee of the National Medical Research Council. All patients signed a written informed consent form prior to inclusion.

### 2.2. Catheter Placement and AF Ablation Procedure

All procedures were performed on uninterrupted vitamin K antagonist (VKA) therapy to maintain a therapeutic INR level between 2-3, which was confirmed on the morning of the procedure in line with recent guideline recommendations [14]. All medications with a potential effect on platelet activity were discontinued for a period of at least three half-lives before the procedure. Transesophageal echocardiography was carried out within 24 hours prior to the procedure in order to rule out the presence of a cardiac thrombus. Procedures were carried out under conscious sedation, using midazolam and fentanyl. Three punctures of the right femoral vein were performed using the Seldinger technique and introducers with side arms were placed in the vein. A multipolar electrode catheter was placed in the coronary sinus and an intracardiac echocardiography (ICE) catheter in the right atrium to guide transseptal puncture and positioning of the ablation catheter with any of the 3 technologies used (see below). Fluoroscopy- and ICE-guided transseptal puncture was performed with a Brockenbrough needle. Immediately after transseptal puncture and preablation blood sample collection from the left atrium (LA), a 150-IU/kg body weight intravenous heparin bolus was given, followed by a continuous infusion to maintain a minimum target ACT level above 300 ms. LA sheaths were flushed continuously with heparinized saline at a steady rate around 30 ml/h, and additional fluid replacement was allowed in case of hypotonia throughout the procedure.

All PVI procedures were performed with any of 3 ablation technologies: 1, PVAC group: phased RF ablation with the second-generation PVAC-Gold catheter; 2, Cryo group: cryoablation with the second-generation Arctic Front Advance catheter; 3, IRF group: focal irrigated radiofrequency ablation with contact force monitoring using a Thermocool, Smarttouch catheter under guidance with the CARTO Merge 3-D electroanatomical mapping system. The choice between these ablation methods was based on operator and patient preference. Regardless of the ablation technology used, procedural endpoint was PVI, defined as PV-LA entrance block verified with pacing maneuvers according to standard practice. Sinus rhythm (SR) was restored by cardioversion at the end of the procedure as needed.

### 2.3. Phased RF Ablation (PVAC Group)

Phased RF ablation protocol at our center and technical details of the 2^nd^-generation PVAC have been described earlier [15, 16]. Briefly, the Mullins sheath was exchanged for a deflectable 12-Fr long FlexCath sheath and advanced into the LA over a 220 cm guidewire. Submerged loading of the circular PVAC-Gold containing 9 electrodes of gold alloy into the introducer as well as continuous flushing of the sheath with heparinized saline was performed to minimize air ingress. Duty-cycled bipolar and unipolar phased RF energy to all or selected electrode pairs was delivered in a temperature-controlled and power-limited fashion (60°C, maximum 10 W) with typical ablation duration of 60 s. Pulmonary veins (PVs) were electrically isolated by targeted ablation of each PV-LA antrum.

### 2.4. Cryoballoon Ablation (Cryo Group)

Our ablation protocol and technical details of the 2^nd^-generation Arctic Front Advance catheter have been described [16, 17]. Briefly, the Mullins sheath was exchanged for the deflectable 12-Fr long FlexCath sheath and advanced into the LA over a stiff long guidewire. The 28-mm Arctic Front Advance balloon was used in all cases. An 8-pole, circular electrode catheter was advanced through the lumen of the cryoballoon and used as a guidewire to cannulate specific side branches of the PVs to allow continuous monitoring of PV electrograms during each freezing cycle. The balloon was manipulated to obtain the possibly most antral position with still a good seal of the vein as assessed by contrast injection and ICE Doppler. Two freezing cycles of 3–4 minutes in duration were usually applied in each PV based on the achieved temperature and the time to PVI. Temperature between −40°C and −55°C was considered as appropriate, and cryoapplication was terminated in case of lower value to minimize the risk of collateral damage.

### 2.5. Point-by-Point Pulmonary Vein Isolation with Irrigated Radiofrequency Ablation (IRF Group)

After 2 separate transseptal punctures, a Mullins transseptal and a 9 F steerable Agilis sheath were placed in the LA. A circular decapolar Lasso catheter and a contact force ablation catheter were advanced through the Mullins and the Agilis sheath, respectively. A 3D anatomical map of the LA was obtained using the Carto Merge System®. Predefined lines for the ablation circles were tagged in the antrum of the PVs. Point-by-point IRF ablation was performed isolating the left and the right PVs in separate circles. RF energy was delivered in power-controlled mode without ramping using 30–35W on anterior segments and 20–25W on posterior wall with an irrigation flow of 17 cc/min. Energy was delivered for 30 s at each site after obtaining a target contact force >6 g. Ablation tags were displayed using Visitag® (Biosense-Webster Inc, Diamond Bar, CA, USA). PVI in each PV was assessed based on signals recorded through electrodes of the Lasso catheter.

### 2.6. Blood Sampling and Laboratory Investigations

Preablation blood samples were taken from the LA through the Mullins sheath immediately after transseptal puncture and removal of the dilator, before the intravenous administration of unfractionated heparin. Postablation blood samples were collected through the LA sheath after removal of the ablation catheter once the last ablation was finished. Forty-five ml blood samples were drawn from which the first 15 ml of blood was discarded in order to exclude intrasheath hemostasis activation [13]. Blood samples were collected into vacutainer tubes (tubes containing 0.109 M sodium citrate), tubes containing CTAD (buffered citrate, theophylline, adenosine, and dipyridamole), and tubes containing no anticoagulant (serum tubes with polymer gel separator, SST; Becton Dickinson, Franklin Lakes, NJ). Anticoagulated blood samples were centrifuged twice at 1500 g, and SST tubes were centrifuged once at 2000 g, at room temperature for 20 min. Plasma and serum samples were stored at −70°C until further analysis. The measurement of plasminogen activator inhibitor-1 (PAI-1) activity was performed from plasma samples anticoagulated with CTAD; besides this measurement, all hemostasis and fibrinolysis tests were performed using citrated plasma. Screening tests of hemostasis (prothrombin time, activated partial thromboplastin time, and thrombin time) were performed from freshly separated plasma samples using routine methods (Siemens Healthcare Diagnostic Products, Marburg, Germany). Commercially available ELISA tests were used to determine PAI-1 activity (Technozym PAI-1 Actibind, Technoclone, Vienna, Austria) and plasmin-*α*2-antiplasmin (PAP) complex (Technozym PAP complex ELISA kit, Technoclone, Vienna, Austria). Factor VIII (FVIII) activity using a chromogenic assay, von Willebrand factor (VWF) antigen level, and D-dimer levels were measured on a BCS coagulometer by standard methods (Siemens Healthcare Diagnostic Products, Marburg, Germany). sVCAM-1 levels were measured from the stored serum samples using ELISA (Quantikine ELISA human sVCAM-1/CD106 Immunoassay, R&D Systems Europe Ltd, Abingdon). Albumin levels of all serum samples were quantified using standard methods (Roche, Basel, Switzerland). All measurements were corrected to albumin levels measured at the time of blood sampling before and after ablation in order to compensate for plasma dilution as a consequence of fluid replacement with saline during markedly different ablation times required by different ablation techniques.

### 2.7. Statistical Analysis

Statistical analysis was performed using GraphPad Prism Software version 5.0 (La Jolla, CA) and the Statistical Package for Social Sciences (SPSS, Release 22.0, Chicago, IL). Normality of the data was evaluated by the D'Agostino and Pearson omnibus normality test. A paired *t*-test or Wilcoxon matched-pairs rank-sum test was applied for comparing results obtained from preablation and postablation intracardiac samples. ANOVA with Bonferroni post hoc test or Kruskal–Wallis test using Dunn–Bonferroni post hoc test was applied for multiple comparisons of unpaired data. Differences between categorical variables were assessed by the *χ^2^* or Fisher's exact test. *p* < 0.05 was considered statistically significant.

## 3. Results

### 3.1. Baseline Patient and Procedure Characteristics

A total of 31 patients were included in the study (Table 1). PVI was performed with phased RF in 7, with cryoballoon in 10, and with point by point IRF ablation in14 patients. Acute PVI was achieved in all PVs in all patients. No difference in baseline characteristics was found between the 3 groups regarding demographics, comorbidities, echocardiographic parameters, and thromboembolic risk. Left atrial access times were significantly longer with IRF ablation. No procedural complication occurred in any of the patients.

### 3.2. Fibrinolytic Parameters

**Figure 1:**
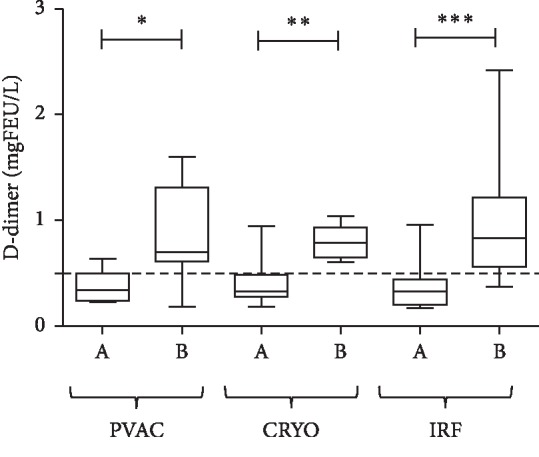
D-dimer levels measured from LA samples before (A) and after (B) PVI with the PVAC, Cryo (cryoballoon), and IRF techniques. Box and whisper plots indicate median, interquartile, and total range. Dashed line indicates the upper limit of reference interval. ^*∗*^*p* < 0.05; ^*∗∗*^*p* < 0.01; ^*∗∗∗*^*p* < 0.001.

Levels of D-dimer in left atrial blood samples increased significantly after ablation with the PVAC (*p*=0.0313), cryo (*p*=0.0078), and IRF (*p*=0.0001; Figure 1). Postablation values exceeded the cutoff value of D-dimer (0.5 mg FEU/L) with all 3 ablation modalities.

**Figure 2:**
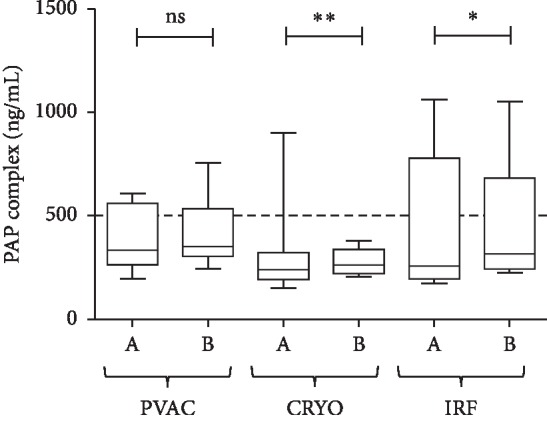
PAP complex levels measured before (A) and after (B) PVI with the PVAC, Cryo, and IRF techniques. Box and whisper plots indicate median, interquartile, and total range. ^*∗*^*p* < 0.05; ^*∗∗*^*p* < 0.01; NS = nonsignificant.

Levels of PAP complex demonstrated no significant change with phased RF ablation (*p*=0.2969), but significantly increased after cryo (*p*=0.0020) and irrigated RF (*p*=0.0166; Figure 2). However, preablation and postablation median PAP values did not exceed the cutoff value proposed by the manufacturer.

**Figure 3:**
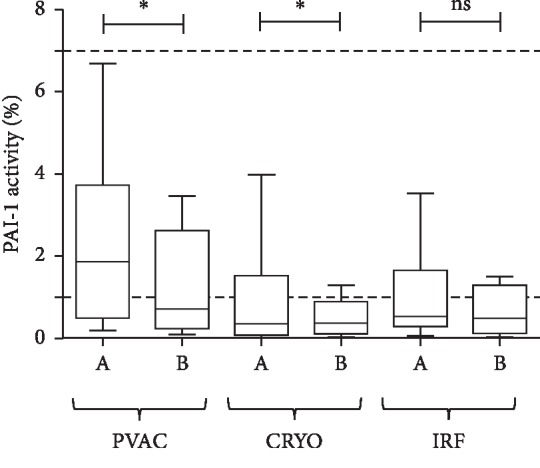
PAI-1 activity measured from LA blood samples before (A) and after (B) PVI with the PVAC, Cryo, and IRF techniques. Box and whisper plots indicate median, interquartile, and total range. ^*∗*^*p* < 0.05.

PAI-1 activity decreased significantly during ablations with the PVAC (*p*=0.0313) and cryo (*p*=0.0313). A nonsignificant trend was observed with IRF (*p*=0.0676; Figure 3).

### 3.3. Parameters Related to Endothelial Damage

**Figure 4:**
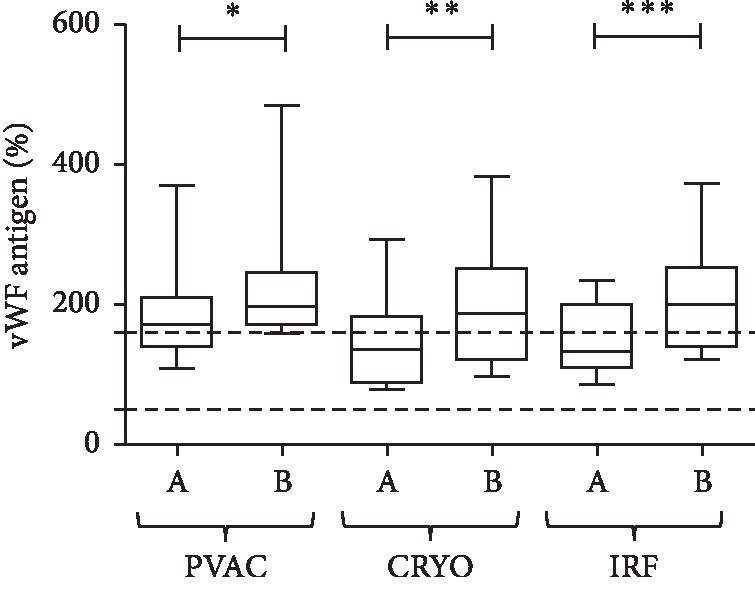
von Willebrand factor antigen levels measured from LA blood samples before (A) and after (B) ablations with the PVAC, Cryo, and IRF techniques. Box and whisper plots indicate median, interquartile, and total range. ^*∗*^*p* < 0.05; ^*∗∗*^*p* < 0.01; ^*∗∗∗*^*p* < 0.001.

VWF antigen levels increased significantly during ablations with all 3 types of catheters. Significant increase was observed after PVAC ablation (*p*=0.0313), after cryo ablation (*p*=0.0039), and a highly significant increase occurred after IRF ablations (*p*=0.0002; Figure 4).

**Figure 5:**
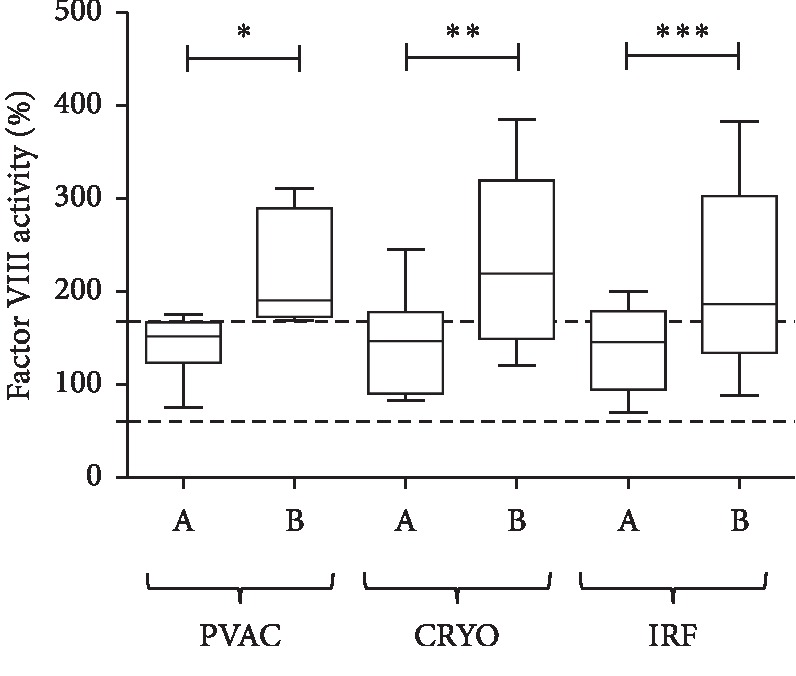
Factor VIII activity measured from LA blood samples before (A) and after (B) ablations with the PVAC, Cryo, and IRF techniques. Box and whisper plots indicate median, interquartile, and total range. ^*∗*^*p* < 0.05; ^*∗∗*^*p* < 0.01; ^*∗∗∗*^*p* < 0.001.

The increase in FVIII activity levels during the ablation procedures was concordant with the increase in VWF antigen levels. A significant elevation in FVIII activity was observed after phased RF (*p*=0.0355), after cryo (*p*=0.0078), and a highly significant elevation was found after irrigated RF ablations (*p*=0.0002; Figure 5).

**Figure 6:**
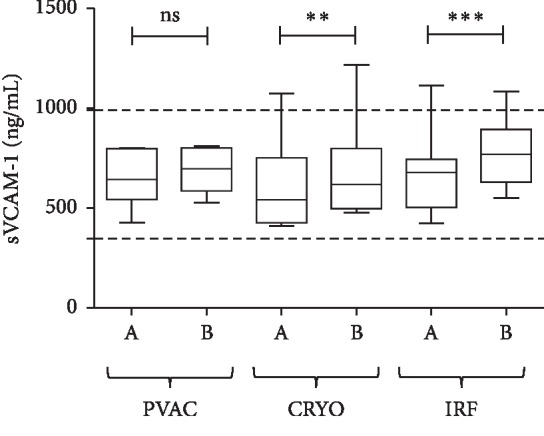
sVCAM-1 levels measured from LA blood samples before (A) and after (B) ablations with the PVAC, Cryo, and IRF techniques. Box and whisper plots indicate median, interquartile, and total range. ^*∗∗*^*p* < 0.01; ^*∗∗∗*^*p* < 0.001.

The levels of soluble VCAM-1 (ng/ml) were not significantly different in the left atrium before and after the PVAC procedure (*p*=0.0963); however, significant increase was detected after cryo (*p*=0.0005) and also after IRF (*p* < 0.0001; Figure 6).

## 4. Discussion

### 4.1. Fibrinolysis Activation during PVI

Limited data and conflicting results are available on hemostasis activation related to invasive electrophysiology procedures. Previous studies evaluated fibrinolysis activation in blood samples obtained from the femoral vein in patients undergoing RF ablation for supraventricular tachycardias (SVTs). Dorbala et al. [10] compared different markers of coagulation and fibrinolytic activation measured in blood samples from the femoral vein obtained immediately after sheath insertion, after a diagnostic electrophysiology study (EPS), and after RF ablation. Significant hemostasis activation was found after EPS as compared to after sheath insertion but no further increase postablation, suggesting that insertion of foreign materials (sheaths, wires, and catheters) in the bloodstream is a significant activator of coagulation. In another report [11] on 37 patients, procedure duration, but not the number of RF ablation, correlated with hemostasis activation. On the contrary, Parizek et al. [9] reported a significant elevation in D-dimer levels after diagnostic EPS and which further increases after ablation. A statistical correlation between *D*-dimer levels and the number of RF applications was also demonstrated. Importantly, well-defined, small-size substrates mainly in the right cardiac chambers were targeted by the ablation in patients who were not anticoagulated before the procedure in these studies.

Data on hemostasis activation related to AF ablation were reported by Bulava et al. [18]: D-dimer levels demonstrated a rapid elevation after sheaths and intracardiac catheter placement with further increase after RF applications, which still persisted 24 hours after ablation. This study was carried out 15 years ago and thereby represents the AF ablation routine of that time: nonirrigated focal RF lesions were placed around or inside the PVs, vitamin K antagonist was interrupted using low molecular weight heparin bridging before the procedure, and intraprocedural heparin applied according to activated partial thromboplastin time (aPTI). Of note, blood samples in this investigation were also obtained from the femoral vein.

As suggested by the results of these studies, insertion of any foreign material into the blood stream initiates hemostasis activation. Previously, our group measured hemostasis activation in blood samples obtained sequentially from different sites including the femoral vein, the left atrium, and the left atrial appendage without any ablation therapy. In this earlier work, which was completed in a different patient cohort than the present study, we demonstrated that transseptal puncture itself is a significant signal for further hemostasis activation [13]. The main focus of the present study was to evaluate the effect of left atrial ablation per se; therefore, blood samples were obtained from the LA before the 1st and immediately after the last energy application. Importantly, our periprocedural anticoagulation scheme represented a common practice of AF ablation centers based on recent guideline recommendations [14]. This included uninterrupted VKA with no bridging, INR level in the therapeutic range before the procedure, and intravenous administration of unfractionated heparin to maintain the ACT level above 300 sec throughout catheter dwelling in the LA. Despite these measures, significant coagulation activation was observed as indicated by the marked change in the levels of different fibrinolysis markers. Postablation D-dimer levels exceeded the normal cutoff value and elevated in the range usually observed during clinical events related to thrombus formation, such as deep vein thrombosis or pulmonary embolism. The levels of PAP complex increased significantly after ablations with cryo and IRF but not with the PVAC. As another indication of coagulation activation, a significant decrease in PAI-1 activity was measured with phased RF and cryo, and a trend for lower values was detected after IRF ablations. These results suggest that significant coagulation activation during AF ablation may not be prevented with the anticoagulation scheme representing a common practice of these days.

### 4.2. Endothelial Activation

Different ablation technologies and ablation energy sources have different biophysical effect on cardiac tissues. Kuhne et al. [19] measured significantly higher levels of Troponin *T* after PVI using IRF as compared to PVI with the 1^st^-generation cryoballoon. However, other investigations comparing biomarkers of myocardial injury after ablation reported conflicting results. In a multicenter study [20], the highest creatinine kinase MB and troponin I levels were demonstrated after PVI with cryoballoon as compared to IRF with or without contact force and laser ablation, while no difference in high sensitive troponin *T*, microparticles, and high-sensitive CRP was found after PVI with IRF versus with cryoballoon in another work [21]. The effect of various ablation technologies on endothelial activation has not been elucidated.

Endothelial damage is a known prothrombotic mechanism as a component of Virchow's triad. The association between left atrial appendage thrombus formation and endocardial expression of VWF has been described [22]. Further, previous studies [18, 23] demonstrated that VWF remains elevated for 24–48 hours after PVI with nonirrigated RF. Prolonged endothelial dysfunction might explain previous observation that thromboembolism mostly occurs within 48–72 hours after AF ablation in the majority of the cases [2]. We demonstrated a significant increase in vWF antigen and in FVIII activity levels with all 3 ablation methods. Postablation elevation of sVCAM levels was associated with the use of cryoballoon and IRF, but not with the PVAC. Overall, these results suggest that significant endothelial damage is caused by contemporary ablation techniques. This finding highlights the importance of strict and continuous anticoagulation in the postablation period.

### 4.3. Effect of Different AF Ablation Techniques on Hemostasis

Due to the low incidence of thromboembolic events related to AF ablation, no data have been published on the differential risk of this complication with different ablation methods. Multiple groups reported on the incidence of new silent cerebral ischemic lesions (SCI) detected by DW-MRI after ablation, and rates as high as 50% were published by some investigators [7, 24–27]. Although the clinical significance of these usually small-sized white matter lesions remains unknown, they were considered by many as a potential surrogate for a clinical thromboembolic event and thereby used to compare different periprocedural anticoagulation schemes [28] and ablation techniques. A higher rate of SCI with phased RF ablation and the 1^st^-generation PVAC was a consistent finding in multiple studies, while cryoablation appeared to be the safest technique [24, 26, 27]. However, more recent communications demonstrated that phased RF technology became safer after procedural and technical modifications [14, 29–32]. We used state-of-the-art and commonly applied AF ablation methods of these days: the 2nd generation of both the phased RF and the cryoballoon technologies and irrigated RF catheters with contact force measurement for point-by-point focal ablation. Although with minor differences, similar effects on fibrinolytic and endothelial activation were detected with any of them. In line with published data [14, 16], LA access times were significantly longer with focal irrigated RF ablation compared with the single-shot technologies in our study; therefore, the specific contribution of catheter dwelling in the LA versus that of the RF energy applications on hemostasis activation could not be determined. This might be a relevant question with potential implications for future innovations in AF ablation technology. Of note, our ablation strategy included PVI alone, without any other substrate modification technique, which would result in more tissue damage, longer LA access time, and potentially more marked hemostasis and endothelial activation.

### 4.4. Potential Clinical Implications

Our results suggest that antithrombotic treatment before AF ablation with widely used techniques of these days needs to be improved for patients' safety. The use of direct oral anticoagulants as a substitute for uninterrupted VKA might be a promising approach with supporting clinical data already available [32–35]. Future studies on fibrinolysis and endothelial activation related to the administration of these agents might provide further insight regarding the mechanism of silent and clinical embolic events. Measurement of these markers might also be valuable to assess the risk of thromboembolic complications involved with novel ablation technologies.

## 5. Limitations

This was a nonrandomized study and patients were assigned to 1 of 3 ablation methods according to their preference and operator's discretion. However, there were no significant differences in baseline clinical parameters between treatment groups. Uncertainties in the interpretation of fibrinolytic activation due to the longer LA access times with IRF ablation remain as mentioned in the discussion. Our patient population, similar to other publications on hemostasis parameters, was relatively small. This sample size may explain the minor inconsistencies in our results, that although demonstrating similar trends, the change in the level of some biomarkers did not reach the degree of statistical significance. Further, the clinical significance of biomarker levels during AF ablation and their use as surrogates of a clinical thromboembolic event remain speculative.

## 6. Conclusions

This prospective observational study demonstrated that significant fibrinolytic and endothelial activation is associated with currently used popular PVI ablation techniques despite the use of periprocedural anticoagulation directed by recent guidelines. No significant differences related to various ablation technologies could be demonstrated.

## Figures and Tables

**Table 1:** Baseline parameters in patients undergoing pulmonary vein isolation with PVAC, Cryo, and IRF technologies. Continuous variables are expressed as mean ± SD or median (interquartile range). Categorical variables are indicated as number (percentage), unless otherwise stated.

Variables	PVAC (7)	Cryo (*n* = 10)	IRF (*n* = 14)	*p* value
Age (years)	64.4 (59.9–69.4)	58.7 (48.4–65.6)	61.3 (55.3–65.1)	0.377
Male, *n* (%)	5 (71.43)	7 (70.00)	7 (50.00)	0.575
Left atrium size (mm)	43.14 ± 14	42.30 ± 3.30	42.00 ± 4.59	0.315
CHADS2-VASC score (mean)	2.29	1.50	2.36	0.514
INR on the day of the procedure	2.55 ± 0.46	2.35 ± 0.39	2.51 ± 0.45	0.208
LA access time (min)	75.00 (64.00–80.00)	83.00 (56.75–113.30)	150.00 (135.8–167.00)	<0.001

## Data Availability

Full data set on which the study is based on will be available on acceptance of the manuscript at http://en.debkard.hu/intracardiacfibrinolysis.htm.
